# Psychological Factors of Vaccination Intent among Healthcare Providers, Parents, and Laypeople

**DOI:** 10.3390/vaccines11121816

**Published:** 2023-12-04

**Authors:** Kaja Damnjanović, Sandra Ilić, Marija Kušić, Milica Lazić, Dragoslav Popović

**Affiliations:** 1 Laboratory for Experimental Psychology, Department of Psychology, Institute of Philosophy, Faculty of Philosophy, University of Belgrade, 11000 Beograd, Serbia; 2 Laboratory for Experimental Psychology, Department of Psychology, Faculty of Philosophy, University of Belgrade, 11000 Beograd, Serbia; sandra.ilic@f.bg.ac.rs; 3 Laboratory for Research of Individual Differences, Department of Psychology, Faculty of Philosophy, University of Belgrade, 11000 Beograd, Serbia; marija.kusic996@gmail.com; 4 Faculty of Philosophy, University of Novi Sad, 21000 Novi Sad, Serbia; milica.lazic@ff.uns.ac.rs; 5 Independent Researcher, 11000 Beograd, Serbia; popovicteam@gmail.com

**Keywords:** vaccination intention, healthcare providers, parents, laypeople, promotive factors, protective factors, risk factors, vulnerability factors, vaccine decision making, vaccination behavior

## Abstract

The interrelatedness of social-structural aspects and psychological features with vaccination intention provides the context to explore personal psychological features related to vaccination. Specifically, we focused on general decision making and vaccine-related dispositions, and their contribution to the intention to vaccinate, within post-pandemic circumstances, after the imposed possibility of choosing a vaccine brand. Our study aimed to map the function (promotive, protective, risk, vulnerability) of a set of personal psychological aspects in the intention to vaccinate among people holding different social roles regarding the vaccination. We surveyed three samples of people: healthcare providers (HPs), parents, and laypeople, within the post-pandemic context. Negative vaccine attitudes lower intention to vaccinate in all regression models (all βs ranging from −0.128 to −0.983, all *p*s < 0.01). The main results indicate that, regardless of the sample/social role, there is a shared attitudinal core for positive vaccination intention. This core consists of [high] trust in large corporations, government, and healthcare systems, as well as perceived consensus on vaccine safety/efficacy and experience of freedom (protective factors), and [low] vaccination conspiracy beliefs, trust in social media, and choice overload (risk and vulnerability factors, respectively). There are no common promotive factors of intention to vaccinate: for parents, perceived consensus on vaccines, and trust in corporations and the healthcare system, play such roles; for HPs, the experience of freedom is obtained as a unique promotive factor. In contrast, for laypeople, no unique promotive factors were found. Our findings provide insights into the function of psychological factors of vaccination intention across different social roles, particularly healthcare providers, parents, and laypeople, and emphasize the need for tailored immunization interventions in the post-pandemic landscape.

## 1. Introduction

The ability of humankind to develop knowledge for the advancement of society should inevitably lead to better population outcomes, but translating scientific insights into an effective policy is neither simple nor straightforward. Mere prescription of an evidence-based immunization schedule by decision-makers is not effective, as cooperation from vaccine recipients, mostly laypeople, is essential. The distinctive feature of vaccines as a medical procedure is that they act at both the individual and community level, making the decision to vaccinate inherently social [1,2,3]. Both recent data and recent global and local responses to the COVID-19 epidemic confirm that this kind of unanimous cooperation is not easy to achieve [4,5]. Over time, studies repeatedly demonstrated that peoples’ vaccination behavior changes in response to the outbreak of vaccine-preventable diseases [6,7,8,9], creating the following reactive-preventive cycle. The proximity of the disease raises the vaccination rate as people take measures to reduce their susceptibility to the disease. Vaccination rate lowers as soon as the perceived danger passes, so the intention to vaccinate changes depending on the proximity of the disease [10]. This cycle is the costliest pattern to foster pro-vaccine attitudes, leads only to short-term herd immunity, and hinders understanding of outbreak causes and mechanisms. Sustainable vaccination programs require not only policies and scientific support but also sustainable vaccination behavior and intentional decisions, which stem from the social and psychological landscape. This is indeed recognized by the stakeholders who started design communication regarding vaccination [1,11,12,13].

The intention to vaccinate is a precursor of vaccination behavior and critical to the success of vaccination programs. However, intentions are changeable [2,14], multi-determined [15], and rooted in structural and social conditions, psychological dispositions, and experiences of the pandemic. As stated, having vaccines and the support of healthcare providers and scientists is insufficient, because this scientific consensus also needs to be communicated to the vaccine recipients, which turns out to be challenging [5,16], presumably due to differing views on vaccination.

### 1.1. Social Roles Regarding Vaccination

*A person’s* social role in vaccination is closely tied to their vaccination behavior, since it both depends on and influences social aspects. Healthcare providers (HPs) and parents are the two pillars of vaccination programs highlighted by the literature. The role of HPs in facilitating vaccination is vital [17,18,19,20], and, although they can differ in terms of stance toward vaccination [21], the majority have a strong positive consensus [22].

Next to the HPs, parents are instrumental in immunization programs as both vaccine recipients and spokespersons for future generations [19,23]. Parents represent a unique group in this matter because they make decisions on behalf of their children, who are unable to do so. Parents are, hence, both decision-makers and proxy decision-makers. Due to HPs’ high involvement in the child’s health, parental concerns may be overemphasized, or miss being addressed, which leads to lowering parents′ intention to vaccinate both themselves and their children. In sum, vaccinating a child is a socially mandated, highly involving parental decision [15,24].

### 1.2. Psychological Factors of Vaccination Behavior

In addition to structural aspects mentioned earlier, such as social roles, policies, and official requirements, psychological features play a crucial role in vaccination behavior. These factors can be broadly categorized into two groups: (a) factors related to general decision making (e.g., trust-related), and (b) factors specific to vaccine decision making. The first ones are relatively stable psychological features, such as thinking styles [25,26], risk-taking tendencies [13], the sources of information people rely on [5,7,27], and both institutional and interpersonal trust [2,28,29,30,31,32]. Factors specific to vaccine decisions are vaccine hesitancy [8], the feeling of being burdened by the decision [33], freedom of choice [33], perceived social consensus and norms [34,35], and vaccine-conspiracy beliefs [36,37,38], which are especially notable during epidemics [4,39,40].

The most notable frameworks explaining vaccination intention are contemporary integrated behavioral models (IBM), which integrate previous traditional behavioral models, such as the theory of planned behavior [41], the health belief model [42], the theory of reasoned action [43], and social-cognitive theory [44]. IBMs assume intention and behavior are equivalent and postulate attitudes, perceived norms, and personal agency as the main determinants of intention [21,43,44,45,46]. In sum, psychological variables have been empirically considered, and their significance for vaccination intention demonstrated, in and outside of IBMs [23].

### 1.3. Risk, Promotive, Vulnerability, and Protective Psychological Factors of Intention to Vaccinate

Psychological features serve multiple functions concerning the intention to vaccinate as a form of preventive health-related behavior [32,47,48]. They can operate as independent promotive or risk factors when they have a direct positive or negative influence on vaccination decisions. Additionally, they serve as protective or vulnerability factors when they interact with other psychological factors and alter the relationship between those factors and the intention to vaccinate.

### 1.4. Context of Epidemic

As previously mentioned, vaccine behavior largely depends on the perceived proximity of the disease in the epidemic [10]. On the psychological level, this means that the influence of structural (e.g., social roles) and psychological factors on vaccination behavior also changes depending on the epidemic. Moreover, social roles are even more pronounced in this context. This is seen in the fact that HPs and laypeople have different conceptualizations of immunization: for HPs, vaccines are routine medical procedures supported by their professional knowledge, while for laypeople, vaccines represent highly involving health decisions not supported by their professional knowledge [15,49]. Epidemic context adds to the anxiety and shapes the information flow and the perception of danger, and leads to the overload of health-related and vaccination intentions [7,29]. This is especially notable in countries in which citizens can choose the type of COVID-19 vaccine, which are, to this day, Hungary and Serbia, with Serbia being the only country that introduced the full free choice among all available vaccines. The Serbian experience with COVID-19 provides particularly useful insights, as the country was among the first to introduce COVID-19 vaccination, beginning in January 2021. This reactive intervention unfortunately did not overcome the absence of timely vaccine-related education and communication, leading to the vaccination coverage of less than 50% of the total population at the end of March 2022.

### 1.5. The Aim, Rationale, and Hypotheses of the Present Study

**Figure 1:**
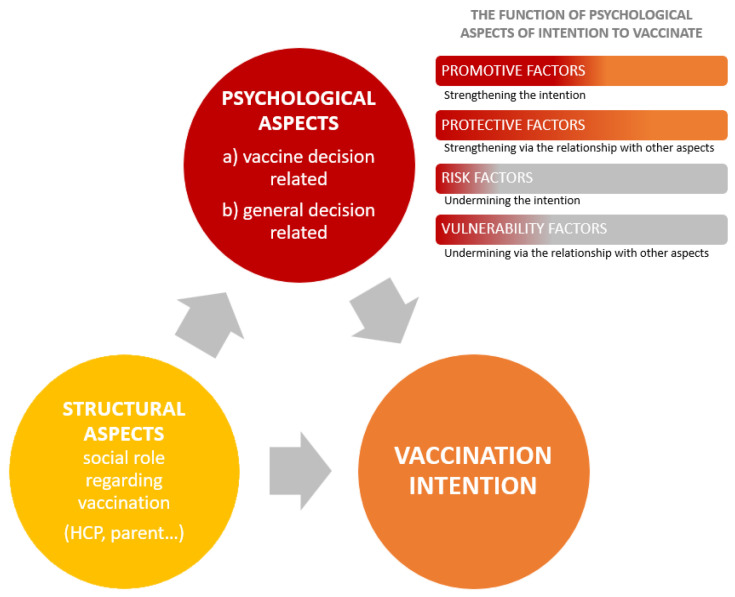
Illustration of interrelatedness of structural and psychological aspects (general and vaccine related) with their possible function in intention to vaccinate, and their contribution to the vaccination intention.

Our study aims to explore psychological features, both general and vaccine-related decision making, and their influence on the intention to vaccinate in three different social-structural roles regarding vaccination: HPs, parents, and laypeople. Figure 1 depicts the complex interaction of (a) social-structural roles, (b) psychological characteristics, and (c) vaccination intention.

People in different social roles regarding vaccination differ in terms of intention to vaccinate. More specifically, their vaccination decision stems from different grounds. For HPs, this is (or should be) knowledge and professional codex [20,22], whereas for laypeople, parents especially, it is based on trust in authorities’ knowledge and their involvement in the decision [2,28,31]. Since psychological aspects could partially be dependent on social roles, it is not obvious whether they have the same function in each group of people. They may serve to promote and protect vaccine intention in one group, and as a risk or vulnerability factor in another group (see Section 1.3). Hence, this study examines the moderating roles of psychological factors in the relationship between negative vaccine attitudes and vaccination intention, in HPs, laypeople, and parents separately.

We expected negative attitudes to be the strongest predictor of vaccination intention in all subsamples. We hypothesized that this relationship would be moderated by psychological factors as follows: the experience of freedom and the high trust in official promoters of vaccines, such as the healthcare system, corporations, and science, would serve as promotive and protective, while vaccine conspiracy beliefs would serve as risk and vulnerability factors in the relationship between negative attitudes and vaccination intention. Since this was not, to our knowledge, previously studied, our study is exploratory, and we have not hypothesized precise expectations regarding possible moderating roles of other variables.

## 2. Materials and Methods

### 2.1. Study Design

A cross-sectional study was carried out between June 2023 and July 2023. We employed a correlational design with negative vaccination attitudes as predictors of the intention to vaccinate one’s (future) child, and psychological dispositions, both general decision-making and vaccine-related, as moderators in this relationship in samples of HPs, parents, and lay people. 

### 2.2. Setting and Sample

The present study was carried out online, via the snowballing method and Facebook advertising. Based on the effect size (*f*^2^ = 0.136; e.g., [50]), the test power of 0.99 and 𝛼 of 0.01 in hierarchical regression analysis with three parameters (predictor, moderator, and their interaction), the sample size to aim for was N = 226 per subsample.

The sample included adult participants from Serbia (N = 745) who belonged to the three different subsamples based on specific inclusion criteria explained in the section below (N_HPs = 219, Mage = 46.31, SD = 10.12, 81.7% women; N_parents = 263, Mage = 41.93, SD = 8.74, 91.3% women, N_laypeople = 263, Mage = 34.93, SD = 9.81, 72.2% women). The detailed socio-demographic structure of the subsamples regarding age, gender, marital status, and socio-economic status (SES) is given in Supplementary Material 1, Table S1, while more specific information about vaccine-type choices is given in Table S2.

### 2.3. Procedure

All participants accessed the questionnaires via the same link, where they were triaged and redirected to a specific questionnaire according to their role relative to vaccination. First, participants were asked if they were healthcare providers in regular contact with patients. If yes, they were redirected to the questionnaire for HPs, and if not, they were asked if they were parents/caretakers of a child, or not. Based on their answers, they were redirected to a questionnaire designed for parents or nonparents (i.e., the laypeople subsample). After accessing the questionnaires that corresponded to the participants’ groups based on parenthood and vocation, all participants included in the final sample gave informed consent to participation. Following this, they completed the questionnaire comprising sociodemographic questions and questionnaires aimed to measure the intention to vaccinate one’s (future) child, and dispositional and vaccine-specific psychological features relevant to vaccination behavior (see Section 2.4).

### 2.4. Materials and Measures

For clarity, we have divided this section into four parts vis-a-vis measures’ conceptual grouping. All the scales used were adapted and translated from English to Serbian via the forward–backward method and were first published in the Damnjanović et al. [3] protocol. The scales, along with the descriptions, scoring, and reliability are given in full in Supplementary Materials 2.

#### 2.4.1. Psychological Dispositions

These dispositions include relatively stable psychological features of a person that may contribute to the formulation and development of vaccination intentions. If not otherwise said, all continuous measurements below were given on a 7-point Likert scale to make these constructs homogeneous, and all scores are calculated as total averages.

*Actively Open-Minded Thinking Scale* (AOT) measures participants’ willingness to change their beliefs in the face of new information, and their general open-minded attitude toward information and reflective belief maintenance thinking [51].

The passive Risk-Taking Scale measures a tendency to passively engage in risks through inaction [52].

*Epistemic Trust Mistrust and Credulity Questionnaire* (ETMCQ) globally measures a person′s trust “in communication and communicated knowledge”, which includes epistemic stances about the quality of information and its sources [53].

#### 2.4.2. Vaccine-Specific Factors

Vaccine-specific factors refer to other psychological features closely tied to, or crucial for, vaccine decision making more specifically realized through the intention to vaccinate one’s (future) child.

*Vaccine Attitudes Scale* measures general positions on vaccines and vaccination behavior [54], consists of 5 items.

*The vaccine Conspiracy Beliefs Scale* measures negative attitudes toward vaccination, and more precisely a tendency to believe in negative vaccine effects and attempts of various institutions (governments, pharmaceutical institutions, scientists) to hide such information [38].

*Experience of Freedom Scale* measures parents’ perceptions of freedom when deciding whether to vaccinate their child(ren) [33].

*The Choice Overload Scale* measures parents’ feelings of informational pressure while making vaccination decisions for their children [33].

*Perceived Consensus and Norms About Vaccination* scale measures perceived scientific consensus about vaccines, as well as vaccination norms in the population. The scale consists of 3 items and is devised for this research, based on Van der Linden’s [55] work on vaccine consensus and norms.

#### 2.4.3. Trust-Related Measures

Trust-related measures pertain to participants’ trust toward different authorities’ knowledge of vaccines. Since measures of trust are based on participant–authority relationships which are shaped by attitudes toward such authorities independently of one’s beliefs about vaccines, we separated these measures into their own category.

*Trust Toward Authorities* [37] scale measures participants’ trust in vaccine knowledge that comes from various sources of authority (corporations, national government, healthcare system, scientists, mainstream media, alternative media, social networks, and their child’s doctor (for parents) or their doctors (for laypeople and HPs).

#### 2.4.4. Vaccination Intention

Vaccination intention refers to the intention to vaccinate one’s (future) child according to the official schedule and is used as a dependent/criterion variable. It is a single-item variable (i.e., “Would you at this time vaccinate your child according to the official vaccination schedule”) expressed on a 7-point Likert scale (1—definitely not; 7—definitely yes).

### 2.5. Data Analysis

As stated, we aimed to examine the complex influence of negative attitudes on vaccination intention by mapping out the moderators of such a relationship in all three subsamples. A series of hierarchical regression analyses with moderation were conducted to achieve this. Negative vaccination attitudes served as predictors, while vaccination intent was the criterion, in all regression models. Other psychological, trust-related, and vaccine-specific variables were thus entered into the model as moderators of this relationship. All variables were centered before creating a product between predictor and moderator variables. The first block of hierarchical regression analysis involved the inclusion of the predictor and moderator variables′ centered values in the model. Subsequently, the interaction between these variables was added in the second block.

## 3. Results

### 3.1. Descriptive Statistics

Descriptive statistics and internal reliability and validity indicators show that we can use the data obtained on these scales in the planned statistical analyses. The observed averages for most scales are around the theoretical averages. The skewness and kurtosis values indicate, however, moderate to high deviations from the normal distribution for most variables in all three samples, except for passive risk-taking, epistemic mistrust, and trust toward government and independent media, which are normally distributed. Epistemic trust, experience of freedom, and choice overload for HPs and lay people sub-samples are also normally distributed. The descriptive statistics, internal reliability, and validity indicators, along with the distribution (a)symmetry values are in Supplementary Material 1.

### 3.2. Do Psychological Dispositions, Vaccine-Specific Factors, and Trust-Related Measures Moderate the Relationships between Negative Attitudes toward Immunization and Vaccination Intention?

A series of separate hierarchical regression analyses were conducted to test the moderating roles of psychological dispositions (passive risk-taking, AOT, epistemic trust, epistemic mistrust, and epistemic credulity), vaccine-specific factors (experience of freedom, choice overload, perceived consensus, subjective norms, and conspiracy beliefs), and trust-related measures (trust in corporations, government, healthcare system, scientists, mainstream media, independent media, and social networks) in the relationship between negative attitudes toward vaccination and vaccination intention on three sub-samples. The following analyses are divided into three organizational units, depending on the group to which the moderator variables belong.

#### 3.2.1. Do Psychological Dispositions Moderate the Relationships between Negative Attitudes toward Immunization and Vaccination Intention?

In all analyses, negative attitudes towards vaccination have moderate to strong negative effects on vaccination intention (all *β*s ranging from −0.531 to −0.849, all *ps* < 0.01). None of the psychological dispositions have significant main effects on vaccination intentions (Table 1).

The negative relationship between negative attitudes towards vaccination and vaccination intention is stronger in conditions of high scores on passive risk-taking in the HPs sub-sample (*β* = −0.115, *p* < 0.05). Similarly, the association between negative attitudes and vaccination intention is stronger under conditions of high scores on epistemic credulity in the parents sub-sample (*β* = −0.079, *p* < 0.05). On the other hand, AOT weakens the association between negative attitudes towards vaccination and vaccination intention in the laypeople sub-sample (*β* = 0.187, *p* < 0.01). However, in addition to the fact that 12 of 15 examined interacting effects are not statistically significant, it should be highlighted that the effect sizes for the significant interactive effects are small.

#### 3.2.2. Do Vaccine-Specific Factors Moderate the Relationships between Negative Attitudes toward Immunization and Vaccination Intention?

All tested models are statistically significant, explaining between 31.4% and 76.6% of the variance in vaccination intention (Table 2). In all conducted analyses, negative attitudes towards vaccination have significant but varying negative effects on vaccination intention (all *β*s ranging from −0.128 to −0.983, all *p*s < 0.01). Conspiracy beliefs have negative main effects on vaccination intention in all three samples. In the HPs sub-sample, the experience of freedom shows a positive correlation with vaccination intention (β = 0.167, *p* < 0.01), while the main effect of perceived consensus on vaccination intention is registered in the parents’ sub-sample (*β* = 0.100, *p* < 0.05).

Regarding interacting effects, the association between negative attitudes and vaccination intention is stronger in conditions of high conspiracy beliefs and choice overload, and weaker in conditions of high perceived consensus and experience of freedom in all three sub-samples.

#### 3.2.3. Do Trust-Related Measures Moderate the Relationships between Negative Attitudes toward Immunization and Vaccination Intention?

All tested models are statistically significant, explaining between 32.4% and 73.8% of the variance in vaccination intention (Table 3). In all conducted analyses, negative attitudes towards vaccination have significant, but varying, negative effects on vaccination intention (all *β*s ranging from −0.184 to −0.853, all *p*s < 0.01). In the parents sub-sample, trust in corporations (*β* = 0.149, *p* < 0.01) and in the healthcare system (*β* = 0.172, *p* < 0.01) have significant positive main effects on vaccination intention. Trust in social networks (β = −0.086, *p* < 0.05), on the other hand, had significant negative main effects on vaccination intention in the sample of laypeople.

In terms of interacting effects, the association between negative attitudes and vaccination intention is weaker in conditions of high scores on trust in corporations, government, and healthcare systems in all three sub-samples. Similarly, in conditions of high trust in scientists, the negative relationship between negative attitudes and vaccination intention is weaker in the sub-samples of HPs and laypeople, but not in the sub-sample of parents. In conditions of high trust in mainstream and independent media, this relationship is also weaker, but only in the HPs sub-sample. Conversely, in conditions of high trust in social networks, the negative association between negative attitudes and vaccination intention is stronger in all three sub-samples.

## 4. Discussion

The present study aimed to investigate different potential moderators (promotive, protective, vulnerability, and risk factors) of the relationship between negative attitudes toward vaccination and vaccination intention in groups with different social roles related to vaccination. Previous findings indicate differences in vaccination attitudes between HPs, parents, and laypeople [19,20,21,23,56,57], but these social roles have not, to our knowledge, been compared relative to the factors that might influence the effects of vaccination attitudes. The results of the present study revealed which dispositional, vaccine-specific, and trust-related factors exert influence on either the vaccination intention (promotive and risk factors) or the “negative attitudes—vaccination intention” relationship (protective and vulnerability factors). We first discuss results relative to the commonality of these effects (direct and moderating) between groups.

### 4.1. Similarities between Parents, Healthcare Providers, and Laypeople

**Figure 2:**
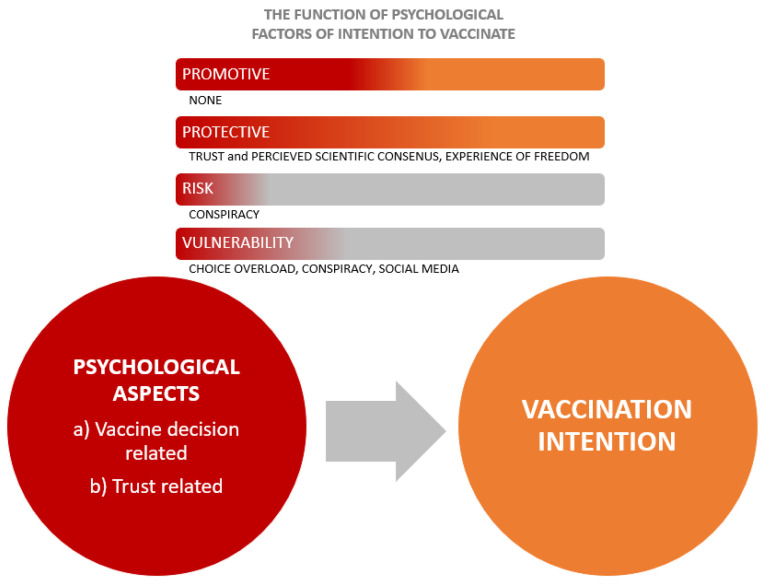
Functions of vaccine-related and trust-related psychological aspects in shaping vaccination intention.

In all three samples, conspiracy beliefs decrease vaccination intention (risk factor), and strengthen the relationship between negative attitudes towards vaccination and vaccination intention (vulnerability factor), confirming the findings of the previous studies [36,37,39,58,59,60]. Choice overload also represents a vulnerability factor for the intention to vaccinate in all three sub-samples. If one perceives there are better and worse options, and worries about making a “bad” choice, they delay immunization [61]. Figure 2 below shows a visual summary of our findings relative to the common promotive, protective, risk, and vulnerability psychological factors that shape vaccination intention in all three samples.

Similarly, trust in social networks moderates the relationship between vaccination attitudes and intention, which is expected given that vaccination conspiracy theories and anti-vaccination propaganda are largely disseminated through these channels of communication [7,40]. Conversely, trust in more controlled and formal sources of information i.e., government, healthcare system, and corporations, serves as a common protective factor. This is in line with findings that the level of trust in institutions responsible for disseminating immunization information successfully mitigates the influence of negative attitudes on behavioral intentions and acceptance of formal and informal norms [29,34,35,62,63]. The perception of consensus regarding vaccination among scientists has a protective role relative to vaccination intention in all sub-samples. People who trust their institutions and perceive scientific consensus are more likely to think that official information about the safety and efficacy of vaccines is true, which makes them more resilient to the consequences of negative attitudes toward vaccination [39,62,64].

The experience of freedom serves as a protective factor of intention to vaccinate in all three samples. Participants who perceive themselves or parents in general as having the ability to make a free choice, review information, and think clearly, decide to vaccinate under a weaker influence of negative vaccination attitudes. In short, any medical decision, including the one regarding childhood vaccination, should be informed and not pressured [61], and a cooperative patient–physician relationship should be aimed for [65] as it can mitigate the deteriorating effects of negative attitudes regarding vaccination.

### 4.2. Differences between Parents, Healthcare Providers, and Laypeople 

**Figure 3:**
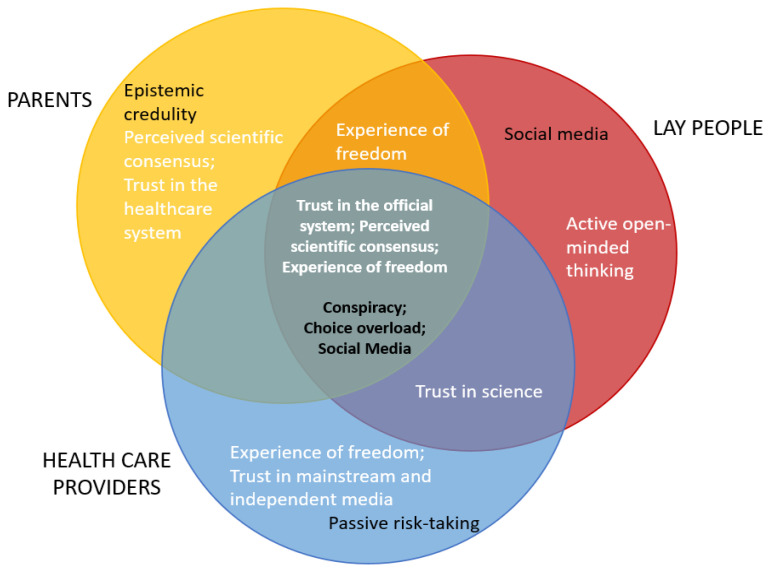
Schematic overview of common and unique protective/promotive (white letters) and risk/vulnerable (black letters) factors in three subsamples.

*For parents*, greater perception of scientific consensus on vaccination, and greater trust in large corporations and the healthcare system, were unique promotive factors of vaccination intention. Although this is in line with previous findings [62,64], the present study shows that these factors have a greater role in the case of parents. Scientific consensus has a direct positive influence on parents’ vaccination intention but also mitigates the effects of negative vaccine attitudes. Figure 3 represents a further visual illustration of the results of this study regarding both unique and common factors that shape vaccination intention in all three samples.

Parents’ trust in institutions, specifically corporations, and the healthcare system, leads to acceptance of vaccination norms those institutions prescribe, e.g., intention to vaccinate, which is corroborated in previous research as well [63]. Unique to parents is the finding that trust in science doesn’t affect vaccination intention, nor does it influence the relationship between negative attitudes and intention to vaccinate a child. So, parents’ positive behavior regarding childhood vaccination is influenced mostly by readily available official information, i.e., tertiary literature about vaccines. Specific to parents also was the role of epistemic credulity as a vulnerability factor. This is in line with findings demonstrating a negative association of credulity with vaccination intention and confidence in the safety of COVID-19 vaccines and discerning false information [53]. In sum, based on our findings, parents’ decision-making process could be relieved with more structured public communication about medical/scientific stances and knowledge of vaccines that emphasizes scientific consensus.

For laypeople, a unique protective factor for the relationship between negative vaccine attitudes and vaccination intention is a greater tendency to revise beliefs considering new information (AOT). This confirms the previously registered positive association of AOT with (positive) vaccine attitudes and vaccination intention, and negative association with misconceptions about vaccines [66]. Trust in social media emerged as a unique risk factor for this group. The fact that trust in social media, in laypeople, serves as both a risk and a vulnerability factor may reflect their greater reliance on such sources of information, which communicate medical information unclearly and disseminate conspiratorial narratives [4,37,40,66]. However, trust in scientists was a protective factor for this subsample.

Finally, only for healthcare providers, the experience of freedom emerged as a promotive factor, and passive risk-taking as a vulnerability factor. This reflects differences between HPs’ and laypeople’s conceptualizations of vaccines—while for HPs vaccination represents a routine procedure, for laypeople, including parents, this is not the case, and the “risks” that parents and laypeople take by lacking intention to vaccinate their (potential) children are actively taken risks. Examination of differences in vaccination conceptualization between HPs and laypeople using both active and passive risk-taking measures could be a fruitful future line of research.

Interestingly, trust in both mainstream and independent media represents a unique protective factor in the relationship between HPs’ negative attitudes and vaccination intention. A possible explanation is that HPs, whose opinions are, by vocation, shaped by official expert and scientific influences, can discern between misinformation and facts in such media. So, factual information regardless of its source might positively influence the relationship between attitudes and intention to vaccinate when it comes to HPs. In favor of this interpretation is the finding that exposure to health-related information in mainstream media positively influences vaccination intention in other cultural contexts [67]. This hypothesis should be tested in further research on social roles relative to vaccination intention.

### 4.3. Limitations and Future Directions

Several limitations to this study should be noted. First, none of the three sub-samples were statistically representative of the population they belong to, and the study’s design was cross-sectional, which renders causal inference impossible. Next, our data are restricted to a single country, and cross-cultural data is needed to evaluate the role of psychological, vaccine-specific, and trust-related factors in vaccination intention in different cultures, with different public policies regarding vaccination. Future studies on vaccination intention in populations that differ by social roles relative to vaccination should aim for samples, from different countries, that are more representative and should include more participants who, in some regard, oppose adult vaccination (e.g., vaccination against COVID-19), as well as assess vaccination attitudes toward varying types of vaccines. A longitudinal or repeated-measures design would enable the assessment of changes in attitudes due to changes in the overall climate regarding immunization among adults that manifest as the reactive-preventive pattern. Measuring reasoning-related cognitive constructs, such as omission, hindsight, or outcome bias would enable insight into the characteristics of the immunization-decision-making processes.

## 5. Conclusions

It is of immense importance to map psychological differences and similarities in groups that differ by social role relative to vaccination, especially in the light of sustainable positive discourse regarding vaccination, and with the aim of consensual and shared decision making. The aim of the present study was thus to investigate the interplay between social roles regarding vaccination and the role of psychological, vaccine-specific, and trust-related dispositions in the relationship between negative attitudes toward vaccination and vaccination intention.

Our findings provide insights into the differences between the social roles of HPs, laypeople, and parents regarding immunization relative to factors’ roles (promotive, protective, risk, and vulnerability factors). Custom-made approaches should be devised to mitigate the specific risk or vulnerability factors and make use of protective and promotive factors registered in these different groups.

Equally important, the present study indicates that, regardless of the social role, a shared attitudinal core for a positive stance regarding vaccines includes high trust in the healthcare system, government, and corporations, high perceived scientific consensus, high experience of freedom, low choice overload, low conspiracy belief, and low trust in social networks as a source of information. Considering these data, communication strategies to improve vaccination uptake aimed at all groups should emphasize scientific consensus and aim for clear and structured dissemination of consistent information via both official expert channels of communication and social networks and independent media.

## Figures and Tables

**Table 1:** Psychological dispositions as moderators in the relationships between negative attitudes toward immunization and vaccination intention.

Moderator:	Health Providers			Parents			Laypeople		
	B	S.E	β	B	S.E	β	B	S.E	β
Passive risk-taking	R = 0.565 **, ΔR^2^ = 0.013 *			R = 0.782 **, ΔR^2^ = 0.002			R = 0.835 **, ΔR^2^ = 0.000		
Negative attitudes	−0.755	0.082	−0.531 **	−1.439	0.073	−0.775 **	−1.229	0.055	−0.849 **
Passive risk taking	−0.028	0.081	−0.020	0.014	0.073	0.008	0.054	0.052	0.037
Interaction	−0.137	0.068	−0.115 *	−0.073	0.066	−0.043	0.016	0.054	0.011
AOT	R = 0.556 **, ΔR^2^ = 0.004			R = 0.781 **, ΔR^2^ = 0.000			R = 0.850 **, ΔR^2^ = 0.022 **		
Negative attitudes	−0.776	0.082	−0.546 **	−1.439	0.073	−0.775 **	−1.002	0.063	−0.692 **
AOT	0.005	0.082	−0.004	0.047	0.073	0.025	0.084	0.055	0.058
Interaction	−0.090	0.082	−0.063	0.038	0.067	0.022	0.189	0.042	0.187 **
Epistemic trust	R = 0.558 **, ΔR^2^ = 0.003			R = 0.780 **, ΔR^2^ = 0.000			R = 0.834 **, ΔR^2^ = 0.000		
Negative attitudes	−0.798	0.082	−0.561 **	−1.448	0.074	−0.780 **	−1.201	0.051	−0.829 **
Epistemic trust	−0.079	0.081	−0.056	−0.008	0.074	−0.004	0.018	0.050	0.050
Interaction	0.079	0.084	0.054	0.008	0.063	0.005	0.021	0.045	0.045
Epistemic mistrust	R = 0.564 **, ΔR^2^ = 0.002			R = 0.781 **, ΔR^2^ = 0.000			R = 0.834 **, ΔR^2^ = 0.000		
Negative attitudes	−0.796	0.080	−0.560 **	−1.449	0.072	−0.781 **	−1.211	0.050	−0.836 **
Epistemic mistrust	0.146	0.080	0.103	0.067	0.073	0.036	−0.002	0.050	−0.002
Interaction	−0.063	0.086	−0.042	0.022	0.087	0.010	0.019	0.043	0.043
Epistemic credulity	R = 0.557 **, ΔR^2^ = 0.001			R = 0.785 **, ΔR^2^ = 0.006 *			R = 0.834 **, ΔR^2^ = 0.001		
Negative attitudes	−0.773	0.083	−0.543 **	−1.461	0.072	−0.787 **	−1.207	0.051	−0.831 **
Epistemic credulity	0.078	0.081	0.055	−0.039	0.072	−0.021	0.008	0.052	0.006
Interaction	0.052	0.078	0.039	−0.161	0.078	−0.079 *	−0.041	0.046	−0.031

Note. * *p* < 0.05. ** *p* < 0.01. B represents unstandardized regression weights; S.E represents standard errors; *β* indicates the standardized regression weights.

**Table 2:** Vaccine-specific factors as moderators in the relationships between negative attitudes toward immunization and vaccination intention.

Moderator:	Health Providers			Parents			Laypeople		
	B	S.E	β	B	S.E	β	B	S.E	β
Experience of freedom	R = 0.608 **, ΔR^2^ = 0.036 **			R = 0.788 **, ΔR^2^ = 0.008 *			R = 0.847 **, ΔR^2^ = 0.022 **		
Negative attitudes	−0.564	0.091	−0.397 **	−1.323	0.086	−0.713 **	−1.072	0.058	−0.740 **
Experience of freedom	0.238	0.081	0.167 **	0.085	0.084	0.046	0.055	0.050	0.038
Interaction	0.236	0.068	0.216 **	0.148	0.064	0.102 *	0.242	0.054	0.170 **
Choice overload	R = 0.598 **, ΔR^2^ = 0.048 **			R = 0.792 **, ΔR^2^ = 0.008 *			R = 0.838 **, ΔR^2^ = 0.005 *		
Negative attitudes	−0.548	0.096	−0.385 **	−1.231	0.093	−0.663 **	−1.141	0.057	−0.788 **
Choice overload	−0.126	0.080	−0.089	−0.163	0.069	−0.088	−0.075	0.052	−0.052
Interaction	−0.322	0.081	−0.264 **	−0.166	0.069	−0.116 *	−0.117	0.058	−0.077 *
Perceived consensus	R = 0.678 **, ΔR^2^ = 0.128 **			R = 0.794 **, ΔR^2^ = 0.017 **			R = 0.848 **, ΔR^2^ = 0.023 **		
Negative attitudes	−0.340	0.092	−0.239 **	−1.137	0.107	−0.612 **	−0.913	0.085	−0.621 **
Perceived consensus	0.110	0.083	0.077	0.185	0.085	0.100 *	−0.030	0.066	−0.021
Interaction	0.388	0.054	0.463 **	0.243	0.072	0.171 **	0.207	0.045	0.242 **
Subjective norms	R = 0.560 **, ΔR^2^ = 0.004			R = 0.785 **, ΔR^2^ = 0.005			R = 0.836 **, ΔR^2^ = 0.003		
Negative attitudes	−0.783	0.081	−0.550 **	−1.381	0.078	−0.744 **	−1.424	0.146	−0.983 **
Subjective norms	0.105	0.081	0.074	0.068	0.077	0.037	0.024	0.051	0.017
Interaction	−0.087	0.082	−0.061	0.125	0.065	0.078	0.047	0.029	0.163
Conspiracy beliefs	R = 0.656 **, ΔR^2^ = 0.094 **			R = 0.825 **, ΔR^2^ = 0.039 **			R = 0.874 **, ΔR^2^ = 0.040 **		
Negative attitudes	−0.182	0.119	−0.128 *	−0.582	0.131	−0.314 **	−0.561	0.091	−0.387 **
Conspiracy beliefs	−0.170	0.107	−0.119 *	−0.547	0.106	−0.295 **	−0.318	0.085	−0.220 **
Interaction	−0.307	0.051	−0.466 **	−0.368	0.065	−0.306 **	−0.236	0.036	−0.332 **

Note. * *p* < 0.05. ** *p* < 0.01. B represents unstandardized regression weights; S.E represents standard errors; *β* indicates the standardized regression weights.

**Table 3:** Trust-related measures as moderators in the relationships between negative attitudes toward immunization and vaccination intention.

Moderator (Always TA)	Health Providers			Parents			Laypeople		
	B	S.E	Β	B	S.E	Β	B	S.E	β
Corporations	R = 0.608 **, ΔR^2^ = 0.062 **			R = 0.799 **, ΔR^2^ = 0.024 **			R = 0.846 **, ΔR^2^ = 0.021 **		
Negative attitudes	−0.482	0.101	−0.339 **	−1.181	0.090	−0.667 **	−1.106	0.068	−0.702 **
Corporations	0.159	0.082	0.111	0.283	0.080	0.149 **	0.076	0.055	0.052
Interaction	0.468	0.101	0.317 **	0.393	0.095	0.133 **	0.286	0.065	0.193 **
Government	R = 0.619 **, ΔR^2^ = 0.078 **			R = 0.791 **, ΔR^2^ = 0.015 **			R = 0.852 **, ΔR^2^ = 0.029 **		
Negative attitudes	−0.451	0.103	−0.317 **	−1.204	0.100	−0.649 **	−0.892	0.077	−0.616 **
Government	0.110	0.083	0.077	0.204	0.083	0.110	0.133	0.055	0.092
Interaction	0.438	0.084	0.351 **	0.321	0.099	0.164 **	0.323	0.062	0.251 **
Healthcare system	R = 0.665 **, ΔR^2^ = 0.117 **			R = 0.813 **, ΔR^2^ = 0.037 **			R = 0.859 **, ΔR^2^ = 0.038 **		
Negative attitudes	−0.261	0.104	−0.184 *	−0.882	0.114	−0.475 **	−0.792	0.082	−0.547 **
Healthcare system	0.090	0.089	0.064	0.319	0.090	0.172 **	0.106	0.064	0.073
Interaction	0.390	0.058	0.486 **	0.377	0.070	0.273 **	0.271	0.044	0.306 **
Scientists	R = 0.625 **, ΔR^2^ = 0.070 **			R = 0.786 **, ΔR^2^ = 0.004			R = 0.839 **, ΔR^2^ = 0.007 *		
Negative attitudes	−0.520	0.095	−0.366 **	−1.281	0.101	−0.690 **	−1.112	0.067	−0.768 **
Scientists	−0.079	0.107	−0.055	0.101	0.099	0.054	−0.038	0.078	−0.027
Interaction	0.316	0.064	0.382 **	0.108	0.064	0.088	0.118	0.047	0.132 *
Mainstream media	R = 0.576 **, ΔR^2^ = 0.024 **			R = 0.781 **, ΔR^2^ = 0.000			R = 0.838 **, ΔR^2^ = 0.004		
Negative attitudes	−0.707	0.084	−0.497 **	−1.428	0.083	−0.769 **	−1.160	0.064	−0.801 **
Mainstream media	0.084	0.080	0.059	0.058	0.081	0.031	−0.050	0.053	−0.035
Interaction	0.246	0.088	0.165 **	0.031	0.103	0.014	0.118	0.064	0.078
Independent media	R = 0.569 **, ΔR^2^ = 0.017 *			R = 0.780 **, ΔR^2^ = 0.000			R = 0.836 **, ΔR^2^ = 0.000		
Negative attitudes	−0.783	0.080	−0.551 **	−1.453	0.074	−0.783 **	−1.235	0.055	−0.853 **
Independent media	0.060	0.080	0.042	−0.016	0.073	−0.009	−0.085	0.052	−0.059
Interaction	0.184	0.080	0.129 *	−0.011	0.072	−0.006	−0.004	0.043	−0.003
Social networks	R = 0.586 **, ΔR^2^ = 0.027 **			R = 0.793 **, ΔR^2^ = 0.011 **			R = 0.848 **, ΔR^2^ = 0.013 **		
Negative attitudes	−0.683	0.084	−0.481 **	−1.357	0.073	−0.741 **	−1.185	0.048	−0.819 **
Social networks	−0.139	0.079	−0.098	−0.153	0.071	−0.082	−0.124	0.049	−0.086 *
Interaction	−0.216	0.073	−0.274 **	−0.167	0.059	−0.110 **	−0.144	0.041	−0.116 **

Note. * *p* < 0.05. ** *p* < 0.01. B represents unstandardized regression weights; S.E represents standard errors; *β* indicates the standardized regression weights.

## Data Availability

The data presented in this study are openly available at https://researchbox.org/2030.

## Supplementary Materials

The following supporting information can be downloaded at: https://www.mdpi.com/article/10.3390/vaccines11121816/s1, Supplementary 1: Descriptive statistics, internal reliability, and validity indicators, the distribution (a)symmetry (skewness) and tailedness (kurtosis) values; and Supplementary 2: Descriptions of all scales, scales in full, scoring, and reliability.
