# Supplementary material for: Psychological Factors of Vaccination Intent among Healthcare Providers, Parents, and Laypeople
Source: Vaccines (Basel). 2023 Dec 4;11(12):1816. doi: 10.3390/vaccines11121816 (PMC10748119; doi:10.3390/vaccines11121816)
Supplement: Supplementary file 1 [file vaccines-11-01816-s001.zip › Supplementary Material 1 final 19XI authors.pdf]

Supplementary Material 1

Descriptive statistics, internal reliability, and validity indicators, the distribution (a)symmetry (skewness) and tailedness (kurtosis) values

Kaja Damnjanović*, Sandra Ilić, Marija Kušić, Milica Lazić, and Dragoslav Popović
***Correspondence:** Kaja Damnjanović: **kdamnjan@f.bg.ac.rs**

Table S1. Socio-demographic characteristics of the three sub-samples

		Health providers	Parents	Lay people
N (Women %)		219 (81.7%)	263 (91.3%)	263 (72.2%)
Age	M	46.31	41.93	34.93
	SD	10.12	8.74	9.81
SES	M	3.10	3.44	3.01
	SD	1.73	1.79	1.63
Marital status (%)	Single	15.50	1.90	54.40
	Extramarital union	17.40	11	28.50
	Married	58.40	73.40	16.30
	Divorced/Separated	7.30	12.50	0.80
	Widowed	1.40	1.10	/
Education (%)	Primary school	/	/	1.50
	High school	8.70	16.7	10.60
	Student	/	1.10	13.70
	College/University degree	24.70	47.50	33.50
	Specialist academic or vocational studies degree	33.30	3.80	0.80
	Masters	16.90	25.50	31.60
	PhD	16.40	5.30	8.40
COVID-19 vaccine coverage (%)	First dose	87.70	76.00	81.70
	Second dose	99.50	98	99.10
	Third dose	74.90	64.80	75.60

Note. COVID-19 vaccine coverage: second dose coverage represents the percentage relative to the first dose coverage; third dose coverage represents the percentage relative to the second dose coverage.

Table S2. Choice of COVID-19 vaccine

	First dose			Second dose			Third dose		
	**HPs**	**Parents**	**LP**	**HPs**	**Parents**	**LP**	**HPs**	**Parents**	**LP**
Pfizer-BioNTech	46.60	31.90	37.30	63.50	52.5	58.10	62.30	49.50	63.80
Oxford-Astra-Zeneca	8.20	11.80	14.10	6.30	11.5	12.60	1.60	1.50	0.90
Sinopharm	23.70	24.70	24.30	21.40	23.5	21.40	8.90	7.10	4.20
Sputnjik V	8.70	6.80	5.70	7.30	8.0	6.50	0.50	3.10	3.80
Moderna	0.50	0.80	0.40	1.00	2.5	0.50	1.60	3.60	2.80
Skipped a dose	12.30	24.00	18.30	0.50	2.0	0.90	25.10	35.20	24.40
Total	100%								

Note. HPs = Healthcare providers; LP = Lay people; Percentages of the second dose add up to 100% of participants who received the first dose, and percentages of participants who received a third dose add up to 100% of participants who received a second dose.

Table S3. Descriptive statistics of the three sub-samples

	Health providers						Parents						Lay people					
	**M**	**SD**	**Sk**	**SE**	**K**	**SE**	**M**	**SD**	**Sk**	**SE**	**K**	**SE**	**M**	**SD**	**Sk**	**SE**	**K**	**SE**
Psychological dispositions																		
Passive risk-taking	3.13	0.83	0.08	0.16	-0.49	0.33	3.15	0.84	0.34	0.15	-0.01	0.30	3.18	0.74	0.08	0.15	-0.04	0.30
Actively open-minded thinking	5.67	0.86	-0.97	0.16	1.21	0.33	5.64	0.78	-.87	0.15	0.88	0.30	5.87	0.75	-0.75	0.15	0.45	0.30
Epistemic trust	5.18	0.98	-0.31	0.16	-0.04	0.33	5.17	1.16	-0.69	0.15	0.37	0.30	5.12	1.04	-0.36	0.15	-0.37	0.30
Epistemic mistrust	3.81	1.10	0.03	0.16	0.13	0.33	3.81	1.07	0.14	0.15	0.16	0.30	3.98	1.10	0.28	0.15	0.19	0.30
Epistemic credulity	2.81	1.28	0.89	0.16	0.44	0.33	2.64	1.19	0.95	0.15	0.83	0.30	2.63	1.14	1.01	0.15	1.05	0.30
Vaccine-specific factors																		
Vaccination intention	6.50	1.42	-3.02	0.16	8.01	0.33	6.10	1.86	-1.98	0.15	2.43	0.30	6.79	1.45	-2.62	0.15	6.18	0.30
Negative vaccine attitudes	1.74	1.14	2.27	0.16	5.55	0.33	2.11	1.33	1.58	0.15	1.09	0.30	1.99	1.29	1.84	0.15	3.13	0.30
Experience of freedom	4.12	1.52	-0.03	0.16	-0.54	0.33	5.29	2.03	-.90	0.15	-0.54	0.30	3.81	1.30	0.31	0.15	-0.26	0.30
Choice overload	4.23	1.63	-0.01	0.16	-0.68	0.33	2.31	1.82	1.36	0.15	0.76	0.30	4.22	1.58	-0.11	0.15	-0.62	0.30
Perceived consensus	5.03	1.58	-0.89	0.16	0.34	0.33	4.63	1.81	-0.90	0.15	-0.60	0.30	5.12	1.60	-1.02	0.15	0.29	0.30
Subjective norms	5.47	1.58	-0.87	0.16	0.05	0.33	5.53	1.69	-0.96	0.15	-0.19	0.30	5.22	1.60	-.64	0.15	-0.37	0.30
Conspiracy beliefs	2.19	1.43	1.53	0.16	2.00	0.33	2.55	1.70	0.94	0.15	-0.17	0.30	2.24	1.45	1.55	0.15	1.82	0.30
Trust toward authorities																		
Corporations	2.79	1.63	0.53	0.16	-0.59	0.33	2.39	1.51	0.79	0.15	-0.38	0.30	2.77	1.64	0.64	0.15	-0.46	0.30
Government	3.75	1.92	-0.05	0.16	-1.10	0.33	2.99	1.87	0.47	0.15	-0.95	0.30	3.60	1.87	-0.05	0.15	-1.24	0.30
Health-care system	5.37	1.76	-1.17	0.16	0.54	0.33	4.84	1.93	-0.66	0.15	-0.61	0.30	5.21	1.71	-1.08	0.15	0.32	0.30
Scientists	6.27	1.28	-2.46	0.16	6.58	0.33	5.97	1.41	-1.43	0.15	1.36	0.30	6.14	1.39	-2.02	0.15	3.71	0.30
Mainstream media	2.33	1.43	0.85	0.16	-0.04	0.33	2.13	1.43	1.14	0.15	0.50	0.30	2.57	1.51	0.68	0.15	-0.38	0.30
Independent media	3.39	1.75	0.12	0.16	-0.95	0.33	3.13	1.79	0.20	0.15	-0.94	0.30	3.79	1.70	-0.10	0.15	-0.86	0.30
Social networks	1.99	1.25	1.33	0.16	1.49	0.33	1.98	1.29	1.19	0.15	0.44	0.30	2.00	1.22	1.13	0.15	0.71	0.30

Note. M = Mean; SD = Standard deviation; Sk = Skewness; K = Kurtosis; SE = Standard Error.

Table S4. Internal metric characteristics of scales

	Health professionals				Parents				Lay people			
	α	SEm	H5	KMO	α	SEm	H5	KMO	α	SEm	H5	KMO
Psychological dispositions												
Passive risk-taking	0.75	0.41	0.50	0.80	0.74	0.43	0.52	0.79	0.70	0.41	0.50	0.79
Actively open-minded thinking	0.67	0.49	0.57	0.81	0.63	0.47	0.70	0.79	0.72	0.40	0.70	0.87
Epistemic trust	0.70	0.54	1	0.82	0.73	0.60	1	0.83	0.70	0.57	1	0.82
Epistemic mistrust	0.66	0.64	1	0.82	0.56	0.71	0.68	0.61	0.66	0.64	0.64	0.74
Epistemic credulity	0.82	0.54	1	0.93	0.78	0.56	1	0.89	0.77	0.55	0.57	0.86
Vaccine-specific factors												
Negative vaccine attitudes	0.74	0.58	1	0.87	0.82	0.57	1	0.94	0.84	0.52	1	0.95
Experience of freedom	0.81	0.66	1	0.93	0.92	0.57	1	0.99	0.80	0.58	1	0.90
Choice overload	0.84	0.65	1	0.90	0.89	0.60	1	0.96	0.90	0.50	1	0.97
Perceived consensus	0.89	0.52	1	0.87	0.93	0.48	1	0.95	0.88	0.55	1	0.86
Conspiracy beliefs	0.87	0.52	1	0.97	0.92	0.48	1	0.99	0.93	0.38	1	0.99

Note. α = Kuder-Richardson-Cronbach alpha; SEm = Standard Error of Measurement; H5 = Knezević-Momirović measure of homogeneity; KMO = Kaiser-Mayer-Olkin measure of representativity. Internal metrics were calculated for all scales with more than 1 item.

Table S5. Correlations between study variables – health professionals

	1.	2.	3.	4.	5.	6.	7.	8.	9.	10.	11.	12.	13.	14.	15.	16.	17.	18.	19.
1. Passive risk-taking	1																		
2. AOT	**-0.14**	1																	
3. Epistemic trust	**-0.19**	0.02	1																
4. Epistemic mistrust	0.02	-0.12	-0.06	1															
5. Epistemic credulity	**0.24**	**-0.29**	0.12	**0.48**	1														
6. Vaccination intention	-0.09	0.06	-0.08	0.08	0.06	1													
7. Negative vacc. attitudes	0.12	-0.13	0.03	0.04	0.01	**-0.55**	1												
8. Experience of freedom	-0.12	-0.01	0.01	-0.03	0.01	**0.34**	**-0.32**	1											
9. Choice overload	0.03	0.08	-0.05	**0.18**	0.07	**-0.19**	**0.22**	**-0.19**	1										
10. Perceived consensus	**-0.22**	**0.20**	0.12	0.04	0.02	**0.40**	**-0.46**	**0.29**	**-0.19**	1									
11. Subjective norms	-0.13	0.07	**0.17**	-0.08	-0.04	**0.14**	-0.12	0.13	-0.09	**0.14**	1								
12. Conspiracy beliefs	**0.14**	**-0.24**	-0.01	**0.17**	**0.18**	**-0.51**	**0.70**	**-0.30**	**0.22**	**-0.59**	-0.10	1							
13. Trust in corporations	-0.03	0.10	-0.09	-0.05	-0.09	**0.19**	**-0.26**	**0.14**	**-0.15**	**0.27**	0.03	**-0.37**	1						
14. Trust in government	**-0.19**	0.09	0.01	-0.04	-0.09	**0.22**	**-0.37**	**0.20**	**-0.17**	**0.31**	0.03	**-0.49**	**0.52**	1					
15. Trust in health system	**-0.26**	0.12	0.07	0.01	0.02	**0.42**	**-0.54**	**0.36**	**-0.24**	**0.50**	0.03	**-0.63**	**0.32**	**0.65**	1				
16. Trust in scientists	**-0.18**	**0.27**	0.12	-0.08	-0.02	**0.40**	**-0.53**	**0.26**	**-0.15**	**0.57**	0.07	**-0.59**	**0.24**	**0.38**	**0.70**	1			
17. Trust in msm	-0.03	-0.01	-0.02	-0.04	0.08	0.09	-0.09	**0.15**	-0.08	**0.16**	0.08	**-0.15**	**0.39**	**0.35**	**0.27**	**0.18**	1		
18. Trust in im	-0.03	**0.14**	0.08	-0.09	-0.10	0.05	-0.02	**0.14**	0.01	**0.21**	0.10	**-0.19**	**0.28**	**0.20**	0.12	0.12	**0.52**	1	
19. Trust in socials	0.03	-0.06	0.02	-0.07	-0.03	**-0.18**	**0.14**	0.07	0.03	-0.05	0.09	0.11	**0.17**	0.04	-0.12	-0.10	**0.45**	**0.41**	1

Note: AOT - Actively open-minded thinking; Trust in msm – Trust in mainstream media; Trust in im – Trust in independent media; statistically significant correlations are marked in bold

Table S6. Correlations between study variables – parents

	1.	2.	3.	4.	5.	6.	7.	8.	9.	10.	11.	12.	13.	14.	15.	16.	17.	18.	19.
1. Passive risk-taking	1																		
2. AOT	-0.08	1																	
3. Epistemic trust	**-0.17**	0.12	1																
4. Epistemic mistrust	0.01	**-0.14**	-0.01	1															
5. Epistemic credulity	**0.16**	-0.09	0.11	**0.35**	1														
6. Vaccination intention	-0.11	**0.15**	0.11	0.01	-0.06	1													
7. Negative vacc. attitudes	**0.14**	**-.15**	**-0.14**	0.03	0.04	**-0.78**	1												
8. Experience of freedom	-0.11	0.06	0.06	-0.10	-0.07	**0.43**	**-0.49**	1											
9. Choice overload	0.11	-0.10	-0.07	0.09	**0.12**	**-0.54**	**0.59**	**-0.66**	1										
10. Perceived consensus	**-0.16**	**0.14**	0.03	**-0.15**	-0.12	**0.49**	**-0.56**	**0.38**	**-0.43**	1									
11. Subjective norms	-0.09	0.06	**0.15**	0.03	-0.03	**0.30**	**-0.32**	**0.21**	**-0.25**	**-0.21**	1								
12. Conspiracy beliefs	**0.18**	**-0.23**	-0.06	0.08	**0.15**	**-0.73**	**0.79**	**-0.50**	**0.60**	**-0.58**	**-0.33**	1							
13. Trust in corporations	0.01	0.04	-0.07	0.04	0.03	**0.33**	**-0.33**	**0.24**	**-0.32**	**0.27**	**0.17**	**-0.40**	1						
14. Trust in government	-0.02	**0.12**	0.07	-0.10	-0.08	**0.32**	**-0.35**	**0.29**	**-0.34**	**0.30**	**0.15**	**-0.43**	**0.59**	1					
15. Trust in health system	**-0.22**	**0.15**	**0.16**	**-0.12**	**-0.16**	**0.60**	**-0.66**	**0.53**	**-0.53**	**0.48**	**0.32**	**-0.73**	**0.32**	**0.49**	1				
16. Trust in scientists	-0.18	**0.21**	**0.16**	**-0.17**	**-0.19**	**.55**	**-0.65**	**0.41**	**-0.47**	**0.51**	**0.28**	**-0.66**	**0.23**	**0.30**	**0.67**	1			
17. Trust in msm	0.07	-0.03	0.01	-0.01	0.06	**0.17**	**-0.19**	**0.15**	**-0.15**	**.16**	0.06	**-0.22**	**.41**	**0.44**	**0.27**	0.06	1		
18. Trust in im	-0.08	0.08	**0.14**	**-0.15**	0.05	0.11	**-.15**	0.11	**-.13**	**0.19**	-0.06	**-0.21**	**0.19**	**0.24**	0.10	**0.15**	**0.42**	1	
19. Trust in socials	0.05	-0.12	0.06	0.07	**-0.19**	**-0.21**	**0.16**	-0.11	**.13**	-0.11	-0.11	**0.19**	0.06	-0.05	**-0.17**	-0.11	**0.32**	**0.42**	1

Note: AOT - Actively open-minded thinking; Trust in msm – Trust in mainstream media; Trust in im – Trust in independent media; statistically significant correlations are marked in bold

Table S7. Correlations between study variables – lay people

	1.	2.	3.	4.	5.	6.	7.	8.	9.	10.	11.	12.	13.	14.	15.	16.	17.	18.	19.
1. Passive risk-taking	1																		
2. AOT	**-0.15**	1																	
3. Epistemic trust	0.04	0.05	1																
4. Epistemic mistrust	0.09	-0.02	**-0.16**	1															
5. Epistemic credulity	**0.20**	**-0.17**	**0.16**	**0.37**	1														
6. Vaccination intention	**-0.21**	**0.47**	0.11	-0.09	**-0.17**	1													
7. Negative vacc. attitudes	**0.30**	**-0.49**	-0.11	0.11	**0.21**	**-0.83**	1												
8. Experience of freedom	**-0.15**	0.10	0.04	0.01	-0.01	**0.26**	**-0.30**	1											
9. Choice overload	**0.14**	-0.05	0.09	**0.12**	0.08	**-0.26**	**0.27**	**-0.35**	1										
10. Perceived consensus	**-0.22**	**0.43**	**0.12**	-0.08	**-0.17**	**0.60**	**-0.69**	**0.28**	**-0.21**	1									
11. Subjective norms	**-0.16**	0.10	**0.17**	0.01	-0.02	**0.23**	**-0.26**	**0.26**	**-0.22**	**0.16**	1								
12. Conspiracy beliefs	**0.30**	**-0.48**	-0.08	0.10	**0.20**	**-0.80**	**0.85**	**-0.32**	**0.32**	**-0.69**	**-0.26**	1							
13. Trust in corporations	-0.10	0.10	-0.01	-0.07	0.03	**0.28**	**-0.34**	**0.24**	**-0.13**	**0.26**	**0.15**	**-0.39**	1						
14. Trust in government	**-0.17**	**0.23**	0.05	**-0.21**	-0.11	**0.40**	**-0.45**	**0.22**	**-0.18**	**0.42**	**0.19**	**-0.52**	**0.48**	1					
15. Trust in health system	**-0.22**	**0.44**	0.14	**-0.20**	-0.12	**0.63**	**-0.69**	**0.26**	**-0.26**	**0.64**	**0.25**	**-0.72**	**0.38**	**0.64**	1				
16. Trust in scientists	**-0.31**	**0.41**	0.09	**-0.22**	**-0.27**	**0.57**	**-0.65**	**0.21**	**-0.26**	**0.64**	**0.16**	**-0.70**	**0.36**	**0.45**	**0.73**	1			
17. Trust in msm	**-0.13**	**0.16**	0.06	-0.05	0.06	**0.20**	**-0.30**	0.07	-0.06	**0.33**	0.11	**-0.30**	**0.44**	**0.47**	**0.38**	**0.28**	1		
18. Trust in im	-0.07	**0.14**	0.11	-0.08	-0.04	**0.20**	**-0.30**	-0.01	**-0.14**	**0.34**	0.05	**-0.34**	**0.27**	**0.29**	**0.35**	**0.32**	**0.47**	1	
19. Trust in socials	-0.10	**-0.14**	0.03	-0.06	0.07	**-0.18**	0.09	-0.01	0.07	0.01	**-0.13**	0.10	0.10	0.08	-0.02	-0.08	**0.37**	**0.34**	1

Note: AOT - Actively open-minded thinking; Trust in msm – Trust in mainstream media; Trust in im – Trust in independent media; statistically significant correlations are marked in bold
