# Supplementary material for: Psychological Factors of Vaccination Intent among Healthcare Providers, Parents, and Laypeople
Source: Vaccines (Basel). 2023 Dec 4;11(12):1816. doi: 10.3390/vaccines11121816 (PMC10748119; doi:10.3390/vaccines11121816)
Supplement: Supplementary file 1 [file vaccines-11-01816-s001.zip › Supplementary Material 2 final.pdf]

Supplementary Material 2

Descriptions of all scales, scales in full, scoring, and reliability

Following section *2.4 Materials and Measures* of the main text

Kaja Damnjanović*, Sandra Ilić, Marija Kušić, Milica Lazić, and Dragoslav Popović

***Correspondence:** Kaja Damnjanović: **kdamnjan@f.bg.ac.rs**

Socio-structural variables

Vaccine-facilitating social roles: *parents, lay people, medical workers*

Triage procedure:

1. *Are you a medical worker with patient contact* – yes/no (if yes: medical workers)
2. If-no-then: *Are you a parent* – yes/no (if yes: parents)
3. If-no-then: lay people

Socio-demographic variables

Age (*expressed in Arabic numbers*)

Gender: *Please select your gender:*

a) Woman
b) Man
c) Other

Education: *Please select the highest education level that you completed:*

a) Finished primary school
b) Finished high school
c) Student
d) Finished college or university
e) Finished specialist academic or vocational studies
f) Finished master studies
g) Finished doctoral studies

Marital status: *Please select your marital status:*

a) Not married/Single

b) In a relationship/Extramarital union
c) Married
d) Divorced or separated
e) Widower

Socio-economic status: *To what extent do you find difficulties in making ends meet in your household?*

7-point Likert scale (1 = Not at all; 7 = It's extremely difficult)

The following subsections are numbered according to the numeration in the main text.

2.4.1. Psychological dispositions

Actively Open-Minded Thinking Scale

We used a revised, short 8-item scale (Baron et al., 2015), where higher scores indicate open-minded thinking while lower scores indicate more rigid, belief-preservation thinking. Cronbach's alpha obtained in our study for subsamples of HPs, parents, and laypeople was satisfactory (respectively, $\alpha_1 = .67$, $\alpha_2 = .63$, $\alpha_3 = .72$).

Please, rate to what extent you agree with these statements.

1. Allowing oneself to be convinced by an opposing argument is a sign of good character.
2. People should take into consideration evidence that goes against their beliefs.
3. People should revise their beliefs in response to new information or evidence.
4. Changing your mind is a sign of weakness. (R)
5. Intuition is the best guide in making decisions. (R)
6. It is important to persevere in your beliefs even when evidence is brought to bear against them. (R)
7. One should disregard evidence that conflicts with one's established beliefs. (R)
8. People should search actively for reasons why their beliefs might be wrong.

7-point Likert scale (1 = Completely disagree; 7 = Completely agree=)

Scores: Average total score

*R indicates reverse-coded items

Passive Risk-Taking Scale

The adapted scale for the Serbian context we used consists of 19 items, where high scores indicate a greater tendency toward risks by not taking some action and low scores indicate responsible low-

risk behavior. Cronbach’s alpha obtained in our study for subsamples of HPs, parents, and laypeople was satisfactory (respectively, $\alpha_1 = .75$, $\alpha_2 = .74$, $\alpha_3 = .70$).

To what extent are you likely to behave in the manner described in the following situations?

1. Buy an expensive product (computer, refrigerator) only after comparing prices in several stores. (R)
2. Install an up-to-date anti-virus on my computer. (R)
3. Check the credit card bill in detail every month. (R)
4. Read the fine print on any major document like a lease, an insurance policy, or a loan. (R)
5. Save receipts and warranty documents of major items in an organized fashion. (R)
6. Back up all important files on the computer, including documents, pictures or videos. (R)
7. Save money regularly. (R)
8. Always lock the house door when going to sleep. (R)
9. Buy clothes without trying them on.
10. Immediately go to the doctor's when something in my body is aching or bothering me. (R)
11. Have regular general medical check-ups every one or two years. (R)
12. Get vaccinated for the flu. (R)
13. Drive straight to the auto repair shop when the car makes a strange noise. (R)
14. Ask the person I am dating about his/her sexual history. (R)
15. Buy serious medical insurance when traveling to another country. (R)
16. Always wear a seatbelt when sitting in the back seat. (R)
17. Pay when parking in a parking-pay zone as directed by the parking meter. (R) (adapted for Serbian context)
18. Change some part in the car (filter, strap, etc..) because the mechanic said it was old and due to fail. (R)
19. Go through customs without declaring about goods I am bringing which are supposed to be taxed.

7-point Likert scale (1 = Not likely at all; 7 = Very likely)

Scores: Average total score

*R indicates reverse-coded items

Epistemic Trust Mistrust and Credulity Questionnaire

We used a short 15-item version of the scale which retains three separately scored subscales that refer to specific epistemic stances: Trust, Mistrust, and Credulity (5 items each). High scores indicate a tendency to be adequately open to information and learning (Trust), to be distrustful of any source of information thus tending to reject communicated knowledge (Mistrust), and to be undecided or unselective about the reliability and quality of information either way (Credulity) (Campbell et al., 2021). Cronbach’s alpha obtained in our study for subsamples of HPs, parents,

and laypeople was satisfactory for Trust (respectively, $\alpha_1 = .70$, $\alpha_2 = .73$, $\alpha_3 = .70$) and Mistrust ($\alpha_1 = .66$, $\alpha_2 = .56$, $\alpha_3 = .66$), and good for Credulity ($\alpha_1 = .82$, $\alpha_2 = .78$, $\alpha_3 = .77$).

Please rate to what extent each of these statements is true for you:

1. I usually ask people for advice when I have a personal problem. (Trust)
2. I find information easier to trust and absorb when it comes from someone who knows me well. (Trust)
3. I'd prefer to find things out for myself on the internet rather than asking people for information. (Mistrust)
4. I often feel that people do not understand what I want and need. (Mistrust)
5. I am often considered naïve because I believe almost anything that people tell me. (Credulity)
6. When I speak to different people, I find myself easily persuaded by what they say even if this is different from what I believed before. (Credulity)
7. Sometimes, having a conversation with people who have known me for a long time helps me develop new perspectives about myself. (Trust)
8. I find it very useful to learn from what people tell me about their experiences. (Trust)
9. If you put too much faith in what people tell you, you are likely to get hurt. (Mistrust)
10. When someone tells me something, my immediate reaction is to wonder why they are telling me this. (Mistrust)
11. I have too often taken advice from the wrong people. (Credulity)
12. People have told me that I am too easily influenced by others. (Credulity)
13. If I don't know what to do, my first instinct is to ask someone whose opinion I value. (Trust)
14. I don't usually act on advice that I get from others even when I think it's probably sound. (Mistrust)
15. In the past, I have misjudged who to believe and been taken advantage of. (Credulity)

7-point Likert scale (1 = Not true at all; 7 = Completely true)

Scores: Separate average total scores for Trust, Mistrust, and Credulity subscales

2.4.2. Vaccine-specific factors

Vaccine Attitudes Scale

We coded the items such that higher scores denote negative attitudes toward vaccination, while lower scores indicate positive vaccination attitudes. Cronbach's alpha obtained in our study for subsamples of HPs, parents, and laypeople was good (respectively, $\alpha_1 = .74$, $\alpha_2 = .82$, $\alpha_3 = .84$).

Please, rate your agreement to each of these items on the following scale, where 1 means “I strongly disagree”, and 7 means “I strongly agree”:

1. The risk of side effects outweighs any protective benefits of vaccines.
2. Vaccinating healthy children helps protect others by stopping the spread of disease. (R)
3. I plan to vaccinate my children. (R)
4. Children do not need vaccines for diseases that are not common anymore.
5. Doctors would not recommend vaccines if they were unsafe. (R)

7-point Likert scale (1 = I strongly disagree; 7 = I strongly agree)

Scores: Average total score

*R indicates reverse-coded items

Vaccine Conspiracy Beliefs Scale

We used a short 6-item version of the scale where higher scores indicate conspiratorial beliefs about vaccines, while lower scores indicate the absence of such a tendency. Cronbach’s alpha obtained in our study for subsamples of HPs, parents, and laypeople was excellent (respectively, $\alpha_1 = .87$, $\alpha_2 = .92$, $\alpha_3 = .93$).

Please, rate your agreement to each of this items in the following scale, where 1 means “I strongly disagree”, and 7 means “I strongly agree”:

1. Vaccine safety data is often fabricated.
2. Immunizing children is harmful and this fact is covered up.
3. Pharmaceutical companies cover up the dangers of vaccines.
4. People are deceived about vaccine efficacy.
5. Vaccine efficacy data is often fabricated.
6. The government is trying to cover up the link between vaccines and autism.

7-point Likert scale (1 = I strongly disagree; 7 = I strongly agree)

Scores: Average total score

Experience of Freedom Scale

The scale has 4 items where higher scores indicate greater feelings of freedom of choice, while lower scores indicate feelings of compulsion and lack of freedom in the vaccine decision-making process. Since this scale is intended for assessing parents’ perceptions regarding the vaccination of their children, it was only administered to parents' sub-sample in its original form. We adapted the scale for sub-samples of lay people and HPs, to ask them to assess “how they think parents feel while deciding whether to vaccinate their child(ren)”. Lay people and HPs also rated all items, but

they were formulated as descriptive norms. Cronbach's alpha obtained in our study for subsamples of HPs, parents and laypeople was very very good (respectively, $\alpha_1 = .81$, $\alpha_2 = .92$, $\alpha_3 = .80$).

A) Parents version

When I was making the decision of whether to vaccinate my child/children…

1. I felt very free in this decision.
2. I was able to choose what I wanted.
3. I was able to ponder and think freely and easily.
4. I felt very constricted in this decision. (R)

7-point Likert scale (1 = Definitely not; 7 = Definitely yes)

Scores: Average total score

*R indicates reverse-coded items

B) Lay people & HPs version

When parents are deciding whether to vaccinate their child/children, rate to what extent do they...

1. ... feel very free in this decision.
2. ... are able to choose what they want.
3. ... are able to ponder and think freely and easily.
4. ... feel very constricted in this decision. (R)

7-point Likert scale (1 = Definitely not; 7 = Definitely yes)

Scores: Average total score

*R indicates reverse-coded items

Choice overload Scale

The scale has 3 items where higher scores denote greater informational overload, and lower scores indicate no choice overload. Note that, same as the previous scale, this is intended for parents' assessments. In the same manner, we adapted the scale for lay people and HPs sub-samples to reflect their descriptive norms about the subject. Lay people and HPs thus assessed, for all items, "how they think parents feel while deciding whether to vaccinate their child(ren)", while parents rated the scale in its original form. Cronbach's alpha obtained in our study for subsamples of HPs, parents, and laypeople was very good (respectively, $\alpha_1 = .84$, $\alpha_2 = .89$, $\alpha_3 = .90$).

A) Parents version

When I was making the decision of whether to vaccinate my child/children…

1. I felt overwhelmed by the decision.
2. The choice exhausted me.
3. It was difficult to keep all the relevant information together.

7-point Likert scale (1 = Definitely not; 7 = Definitely yes)

Scores: Average total score

B) Lay people & HPs version

When parents are deciding whether to vaccinate their child/children, rate to what extent do they...

1. … feel overwhelmed by the decision.
2. The choice exhausted them.
3. It was difficult for them to keep all the relevant information together.

7-point Likert scale (1 = Definitely not; 7 = Definitely yes)

Scores: Average total score

Perceived Consensus and Norms About Vaccination Scale

These items are scored as one average total score for consensus and one single-item variable for norms. Cronbach's alpha for the perceived consensus scale obtained in our study for subsamples of HPs, parents and laypeople were excellent (respectively, $\alpha_1 = .89$, $\alpha_2 = .93$, $\alpha_3 = .88$).

1. Is there a consensus among scientists about the safety of vaccines? (Perceived consensus)
2. Is there a consensus among scientists about the effectiveness of vaccines? (Perceived consensus)

7-point Likert scale (1 = No consensus at all; 7 = Total consensus)

3. Is the "vaccination issue" a big thing in your country? (Subjective norms)

7-point Likert scale (1 = Definitely not; 7 = Definitely yes)

Scores: For perceived consensus (items 1 and 2) – average total score; for subjective norms – a single-item variable

2.4.3. Trust-related measures

Trust Towards Authorities Scale

For every authority source, participants rate their trust on a 7-point Likert scale (1 - strongly mistrust; 7 - strongly trust) and items are separately scored as eight single-item variables.

Please indicate the extent to which you trust the following sources to tell the truth about vaccinations:

1. Corporations
2. National government
3. Healthcare system
4. Science/scientist
5. Mainstream media
6. Alternative media
7. Social networks
8. Your child's doctor (parents version)/Your doctor (lay people & HPs version)

7-point Likert scale (1 = Completely mistrust; 7 = Completely trust)

Scores: A single-item variable for every authority

2.4.4. Vaccination intention – dependent variable

Vaccination intention refers to the intention to vaccinate one's (future) child according to the official vaccination schedule. Higher scores thus reflect a greater vaccination intent. For laypeople and HPs, a formulation "regardless of whether you are a parent or not" was added, based on Stojkovic et al.'s (2017) research.

A) *Parents version:* Would you at this time vaccinate your child according to the official vaccination schedule?
B) *Lay people & HPs version*: Would you at this time vaccinate your child according to the official vaccination schedule, regardless of whether you are a parent or not?

7-point Likert scale (1 = Definitely not; 7 = Definitely yes)

Scores: A single-item variable
